# Is every comparison a thief of joy? Polish validation of the Iowa-Netherlands Comparison Orientation Measure and the indirect role of social comparisons in the relationship between emotional stability and the impostor phenomenon

**DOI:** 10.1371/journal.pone.0333095

**Published:** 2025-09-25

**Authors:** Zuzanna Schneider, Aleksandra Żenda, Natalia Dziura, Paulina Wardawy-Dudziak, Dagna Kocur, Edyta Charzyńska

**Affiliations:** 1 Institute of Psychology, Faculty of Social Sciences, University of Silesia in Katowice, Katowice, Poland; 2 Institute of Pedagogy, Faculty of Social Sciences, University of Silesia in Katowice, Katowice, Poland; Aalborg University, DENMARK

## Abstract

Social comparisons play an integral role in shaping people’s self-perceptions and how they view their accomplishments. The first purpose of our research was to introduce a Polish version of a social comparison measure. The second aim of our studies was to examine the indirect effect of frequent social comparisons — both ability and opinion-related — in the relationship between emotional stability and the impostor phenomenon (IP). Through two studies with a total of 1,466 participants we examined the factor structure of the Polish Iowa-Netherlands Comparison Orientation Measure (INCOM-PL). The results of the confirmatory factor analyses (CFA) supported a two-factor solution of ability and opinion comparisons. The scale showed scalar measurement invariance between men and women. In Study 1 (*N* = 465), we validated the INCOM-PL and found that social comparisons negatively correlated with age and self-compassion, and positively with maladaptive perfectionism. In Study 2 (*N* = 1,001), we found an indirect effect of ability, but not opinion comparisons, in the relationship between emotional stability and IP. This suggests that people low in emotional stability tend to frequently compare their abilities with others, which in turn may reinforce their self-perceived intellectual phoniness. Conditional process analysis revealed that these effects were independent of participants’ gender. Our findings point to a potentially promising direction of targeting maladaptive patterns of social comparisons in interventions aimed at reducing the IP.

## Introduction

Social comparisons, defined as the evaluation of oneself in relation to others, lie at the heart of how humans think, feel, and behave. They provide people with crucial self-relevant information, enabling them to better understand their abilities, opinions, and limitations in the context of others’ achievements and characteristics [1,2]. Comparing oneself with others may serve such purposes as gaining information about one’s social status, enhancing one’s mood, or playing an adaptive role when facing challenging circumstances [3]. More generally, this evaluative process represents the fundamental human desire toward self-understanding [4] and gratifies major motives of self-assessment, self-enhancement, and self-betterment [5–7].

Social comparisons can be categorized into upward and downward comparisons. While upward comparisons involve evaluating oneself against individuals perceived as superior in a specific domain, downward entail comparisons with individuals perceived as inferior in this particular area [8,9]. They can also be divided into two main categories based on what the comparative process refers to: abilities or opinions. Ability comparisons provide insight into how an individual performs relative to others, whereas opinion comparisons help individuals understand commonly held beliefs on specific topics and compare them with their own [4]. The two comparison orientations also differ in their foundations — while ability comparisons are rivalry-based, opinion comparisons are non-competitive and focus on determining how one’s views refer to those of others’ [10,11]. Although the urge to compare oneself with others is universal, the frequency, nature, and consequences of social comparisons vary among individuals because of the underlying personal and situational factors [12].

Certain personality traits may predispose individuals to engage in more frequent social comparisons. In particular, neuroticism is strongly associated with a greater tendency to engage in social comparisons [8,13]. Additionally, a greater tendency to compare oneself with others is associated with lower self-esteem, though the associations between both neuroticism and low self-esteem and social comparisons are driven rather by comparisons of abilities than those of opinions [13]. Furthermore, high neuroticism also predicts a more positive affective response to downward social comparison, suggesting that people low in emotional stability (i.e., high in neuroticism) with low self-esteem may benefit more from information that someone else is even worse-off [14]. Interestingly, while people with high self-esteem are more likely to engage in downward comparisons, those with low and unstable self-esteem are more inclined to engage in upward comparisons [14]. Similarly, there are certain traits that may prevent people from frequently engaging in social comparisons. One such trait is self-compassion — defined as a positive and non-judgmental attitude toward self [15] — as people with high self-compassion are less self-critical and they no longer need to feel superior to others to enhance or defend their own self-esteem. Self-compassion is not only associated with less frequent social comparisons [16], but people with high self-compassion also experience less negative feelings after encountering social comparison [17].

Social comparisons are a complex mechanism that can result in both positive and negative outcomes [8,18]. They can enhance self-esteem, increase motivation, and help adapt to challenging situations. Even though comparisons – especially those in relation to a subjectively superior individual – can cause feelings of envy or inadequacy [19], if done in a constructive manner, they can also serve as inspiration for personal growth and attainment [20]. Nonetheless, this boost of motivation gained through upward comparisons is not endless either and tends to be more beneficial in the short rather than the long term [21]. Notably, there are also risks in frequent social comparisons. Namely, social comparisons tendencies are linked to a variety of negative emotional states, including guilt, envy, regret, defensiveness [22], social anxiety [23–25], or perfectionism [26,27]. Highly perfectionistic people not only tend to compare themselves with others more, but also tend to make less favorable comparisons, that is, perceive themselves as inferior in relation to others [28]. Social comparison-related rumination – that is repetitive thoughts about social comparisons’ outcomes – is associated with perfectionism, burnout, fear of negative evaluation, and depression [29]. Furthermore, frequent social comparisons are also linked to the impostor phenomenon (IP) [30,31] described as subjective experience of intellectual fraudulence and accompanied by a sense that one is less intelligent than others might think [32]. People with high levels of IP tend to compare themselves with others more and when doing so, they focus on exaggerating their own flaws and minimizing them in others [33]. Interestingly, people high in IP indeed compare their abilities with others more, but not their opinions [34]. This distinct mechanism of varying ability- and opinion-related comparisons’ correlates seems to be a persistent pattern also in regard to other mental-health related issues. Ability, but not opinion comparisons, are also related to social anxiety [33], low self-esteem [34], negative social adaptation [35], or experiencing depression and envy [36]. These findings point to the unique nature of both ability- and opinion-related comparisons, which may often be overlooked, when focusing solely on the general tendency to compare oneself with others [37].

The purpose of the current studies is twofold. First, we seek to prepare a valid, reliable, and gender-invariant Polish version of the Iowa-Netherlands Comparison Orientation Measure (INCOM) developed by Gibbons and Buunk [13]. The INCOM has since been adapted to many languages including German [38,39], Spanish [40], Portuguese [41], and Brazilian Portuguese [42], but not yet into Polish. The measure assesses social comparison orientation, that is the tendency to compare one’s abilities and opinions with others originally described by Festinger [4]. Although in their original studies, Gibbons and Buunk [13] claimed viability of a general social comparison score, mainly based on a high intercorrelation of the two ability- and opinion-related factors, a two-factor model outperformed the single-factor solution [13]. More recent studies also did not replicate the high intercorrelation between ability and opinion comparisons but rather showed either a moderate [34,37] or a weak one [11,35,43], suggesting that their variance is largely non-overlapping. Furthermore, the two factors demonstrate different psychological correlates [33–36] and mechanism [10], further supporting their distinction. Therefore, in line with previous research [11,32,33,37], we expect to replicate the two-dimensional model of ability and opinion comparison. Moreover, we aim to test the Polish INCOM’s (INCOM-PL) measurement invariance (MI) between genders, to examine whether the construct is psychometrically equivalent in these groups and, thus, whether their results are comparable.

We seek to validate the Polish INCOM by replicating its previously established correlates: self-compassion, perfectionism in its adaptive and maladaptive form, and age. In line with previous research [16], we expect that social comparisons are negatively related to self-compassion as people high in self-compassion are less likely to evaluate their worth relative to others [15]. However, we presume the correlation with ability comparisons to be stronger than with opinion comparisons, given that only the former is competition-oriented and linked to mental health concerns [44]. Additionally, considering the previously found link between social comparisons and perfectionism [26–28], we expect that social comparisons — especially ability comparisons — will correlate negatively with maladaptive perfectionism characterized by overly high expectations and self-imposed pressure [45]. Lastly, we expect that the tendency to engage in both ability and opinion comparisons lowers with age as young adults tend to be less self-confident and more conforming and thus, more inclined to self-assess in relation to their others [46,47].

The second purpose of our research was to explore the potential indirect effect of social comparisons in the relationship between neuroticism and the IP. People low in emotional stability (or high in neuroticism) are more likely to engage in frequent social comparisons [13,48] and have high levels of the IP [49,50]. Comparing oneself with others is also considered a hallmark trait of those with high levels of IP [51] and the two constructs are linked with one another in both academic settings [52] and online ones through social media sites [53]. People high in neuroticism (or low in emotional stability) also tend to experience more negative consequences of the comparisons they engage in as they are more likely to view themselves unfavorably as a result of this comparative process [48]. They are more likely to direct their comparisons upward and through this comparative lens they often tend to magnify their own self-perceived shortcomings [54]. We presume that individuals with low levels of emotional stability through these frequent, disadvantageous, and often unattainable comparisons reinforce their sense of inadequacy and, consequently, perceive themselves as fraudulent and out of place. Based on these premises, we hypothesize that people with low emotional stability frequently compare themselves – namely their abilities – with others and subsequently experience higher levels of the IP. Therefore, we aim to examine a potentially intervening role of social comparisons — both abilities and opinions — to gain better insight into the underlying mechanisms in these relationships. We expect that the links between emotional stability and IP, and social comparisons and IP may be stronger for women than for men, given that women not only demonstrate higher levels of neuroticism [55], but are also more susceptible to stress, which in turn may drive them to engage in more frequent comparisons [46]. They are also more self-critical [15,56] and more prone to self-derogation [57] thus, can be more at risk of experiencing the negative outcomes of this evaluative process such as stronger feelings of inadequacy resulting in higher levels if IP. For these reasons, we also plan to examine a potentially moderating role of gender in the relationship between emotional stability and the IP through social comparisons.

## Methods

### Procedure

Both studies were conducted online using the LimeSurvey platform. The participants were recruited using snowball sampling method and the invitations to participate in the studies were posted on various social media platforms. The data were collected in Poland between March 7 and June 4, 2022, as part of a larger research project on social comparisons and the impostor phenomenon. The participants’ inclusion criteria in both studies were speaking Polish, a minimum age limit of 18 years, and passing the attention check (participants were asked to select a particular response category, e.g., “strongly disagree”). All participants gave their written informed consent in the online form before completing the study. As a compensation for their participation, after completing the study, participants could voluntarily enter a prize drawing of five bookstore gift cards by signing up in a separate form. Ethical approval of the Ethics Committee at the University of Silesia in Katowice was obtained (KEUS 215/01.2022/W) and the study was conducted in accordance with the Declaration of Helsinki. The Iowa-Netherlands Comparison Orientation Measure is an openly available measure, nonetheless, its authors granted their permission for the translation and further use of the tool.

### Statistical analyses

In Study 1, we started the analyses by conducting a confirmatory factor analysis (CFA) of the INCOM-PL with a robust maximum likelihood estimator (MLR) of the INCOM to determine whether the previously established two-factor structure is replicable in the Polish version of the tool. We followed the recommended use of MLR estimator when the response number is ≥ 5, the thresholds are approximately symmetrical, and the normality assumption is not severely violated [58], which was also previously adopted in other adaptations of the INCOM [39]. We adopted the following goodness of fit criteria to indicate a good fit the model: χ^2^ divided by the degrees of freedom (χ^2^/*df*) ≤ 3; the root mean square error of approximation (RMSEA) <.06, the standardized root mean square residual (SRMR) <.08; the comparative fit index (CFI) and Tucker-Lewis Index (TLI) ≥.95 [59,60]. The following indices indicated an acceptable fit: χ^2^/*df* < 5, CFI and TLI ≥ .90, RMSEA and SRMR ≤ .08 [61,62].

We then tested the INCOM-PL’s measurement invariance (MI) between genders by performing a multi-group CFA (MGCFA) using an MLR estimator. The measurement invariance was determined by testing and comparing subsequently more constrained models: configural (equivalent factor structure), metric (factor loadings fixed to be equal), and scalar (item intercepts fixed to be equal) MI. The following cut-off criteria of changes in subsequent models’ fit (∆) were used to determine whether MI was established: ∆CFI and ∆TLI ≤ −.01, ∆RMSEA ≤ .015, and ∆SRMR ≤ .030 for metric invariance or ≤.015 for scalar invariance [63–65].

At the next step, we calculated the internal consistency of the INCOM-PL’s subscales using Cronbach’s alpha and McDonald’s omega. We then tested the Polish INCOM’s convergent and discriminant validity by correlating its scores with maladaptive and adaptive perfectionism, self-compassion, and age using Pearson’s *r*. Additionally, we used Fisher’s *z* to compare the differences in correlation strength of ability and opinion comparisons.

In Study 2, we reexamined the INCOM-PL’s factor structure using and MI between genders to further validate its psychometric properties and test whether the results of Study 1 are replicable in another sample. In both CFA and MGCFA we used the MLR estimator and the same cut-off criteria as in Study 1. Using Pearson’s *r*, we also conducted a correlation analysis to further test INCOM-PL’s validity and examine its correlations with other variables in the study, namely age, the Big Five personality traits, self-esteem, and the IP. We used the JASP statistical software version 0.95.1 [66] for all the statistical analyses except for the indirect effect models. The CFA and the MGCFA were conducted using the structural equation module (SEM) in JASP.

Lastly, we tested an indirect effect model using the PROCESS_v4.2_beta plug-in [67] in IBM SPSS (version 29.0). We tested a parallel indirect effect model with social comparisons of abilities and opinions as the intervening variables in the relationship between emotional stability and the IP (Model 4). The significance of the indirect effect in the indirect effect model was determined by examining whether the 95% confidence intervals do not contain zero values. Following the recommendation of Abrar et al. [68] we reported the standardized regression coefficients. To evaluate the effect size of the indirect effect, we used the completely standardized indirect effect (*ab*_*cs*_) [69]. Following the recommendation of Kenny [70], we assumed that effect size of.01 would indicate a small indirect effect,.09 a medium one and.25 a large indirect effect. Finally, using a parallel conditional process analysis (Model 59) in the PROCESS plug-in, we tested whether these indirect effect models would be moderated by gender. Given the wide age range in our samples and that people’s tendency to engage in social comparison lowers with age [47], we controlled for participants’ age in both the indirect effect and the conditional indirect effect model.

## Study 1

### Participants

Four hundred and sixty-five adult participants (355 female, 100 male, 8 non-binary persons, and two persons identifying as “other” gender) aged 18–80 years (*M* = 29.33; *SD* = 9.21) took part in the study. Most of them had higher education (62.4%), 36.1% had high-school education, 0.4% had middle-school education, 0.2% had primary education, and 0.9% had vocational education. Most of the participants were working professionals (53.1%). The rest reported their occupations as follows: university students (26.7%), school students (1.7%), working and studying simultaneously (14%), unemployed (3.2%), retired (0.4%), receiving a pension (0.6%). One person did not declare their occupational status (0.2%).

### Measures

To measure social comparisons, we used the Iowa-Netherlands Comparison Orientation Measure (INCOM) [13]. The scale was translated into Polish by three independent translators (a bilingual person, an English philologist, and a psychologist fluent in English), back-translated by two English philologists, and the translations were evaluated by two psychologists fluent in English [71]. The measure consists of 11 items and the scores are summed for indices of two subscales: ability comparisons and opinion comparisons. Two of the items (Item 5 and Item 11) have reversed scoring. Participants rate how much they agree with such statements as “If I want to find out how well I have done something, I compare what I have done with how others have done” or “If I want to learn more about something, I try to find out what others think about it” using a scale from 1 = *strongly disagree* to 5 = *strongly agree*. In the current study the internal consistency coefficients of the full 11-item version of the INCOM were α = .89, ω = .89 for ability comparisons, and α = .64, ω = .68. for opinion comparisons.

We used the Adaptive and Maladaptive Perfectionism Questionnaire (KPAD) [72] to measure the two facets of perfectionism. The scale consists of 35 items and scores are summed for indices of adaptive and maladaptive perfectionism. Participants rate how much they agree with such statements as “I set high standards for myself at work/university” or “I only accept myself when I do everything flawlessly” using a scale from 1 = *strongly disagree* to 7 = *strongly agree*. The reliability in the current study was as follows: adaptive perfectionism (α = .90, ω = .89), maladaptive perfectionism (α = .96, ω = .96).

For measuring self-compassion, we used the Polish version [73] of the Self-Compassion Scale (SCS) [74]. The scale consists of 26 items that can be summed to a general score or six subscales: self-kindness, self-judgment, common humanity, isolation, mindfulness, and over-identification. Participants rate how much they agree with such statements as “I’m kind to myself when I’m experiencing suffering” or “When I’m going through a very hard time, I give myself the caring and tenderness I need” using a scale from 1 = *almost never* to 5 = *almost always*. In the current study the internal consistency coefficients for the total score were α = .95, ω = .95 and for the subscales as follows: self-kindness (α = .90, ω = .90), self-judgment (α = .86, ω = .86), common humanity (α = .83, ω = .83), isolation (α = .79, ω = .79), mindfulness (α = .79, ω = .79), and over-identification (α = .75, ω = .76).

### Results

### CFA and MGCFA

We first tested the originally proposed two-factor model with ability comparisons consisting of six items (Items 1–6) and opinion comparisons consisting of five (Items 7–11) [13]. While the goodness of fit indices indicated an acceptable fit (χ^2^ = 143.4, *df* = 43, *p* < .001, χ^2^/*df* = 3.33, CFI = .94, TLI = .92, RMSEA = .071, RMSEA CI 90% [.059;.083], SRMR = .086), the reversed Item 11 had a factor loading of only .05 (Table 1). We checked the modification indices (MI), which indicated a high cross-loading of the Item 11 with the ability comparison factor (MI = 50.41). The examination of the modification indices also revealed high residual errors between Item 7 and a few of the other items: Item 8 (MI = 51.52), Item 9 (MI = 11.92), and Item 11 (MI = 8.58). It also cross-loaded the ability comparison factor (MI = 11.72).

**Table 1:** Confirmatory factor analysis (CFA) and factor loadings of the Polish version of the INCOM (INCOM-PL).

Construct	Item’s number	Item’s content	Factor loading (λ)	
			Study 1	Study 2
Ability comparisons	1	I often compare how my loved ones (boy or girlfriend, family members, etc.) are doing with how others are doing.	.53	.50
	2	I always pay a lot of attention to how I do things compared with how others do things.	.84	.76
	3	If I want to find out how well I have done something, I compare what I have done with how others have done.	.77	.72
	4	I often compare how I am doing socially (e.g., social skills, popularity) with other people.	.78	.78
	5	I am not the type of person who compares often with others. (reversed item)	.84	.83
	6	I often compare myself with others with respect to what I have accomplished in life.	.79	.75
Opinion comparisons	7*	I often like to talk with others about mutual opinions and experiences.*	.56	−
	8	I often try to find out what others think who face similar problems as I face.	.77	.54
	9	I always like to know what others in a similar situation would do.	.72	.78
	10	If I want to learn more about something, I try to find out what others think about it.	.64	.62
	11*	I never consider my situation in life relative to that of other people. (reversed item)*	.05	−

*Note.* *items were not included in the INCOM-PL. Factor loadings are standardized. Polish version of the scale can be found in S3 File.

These two items also proved problematic in other language versions of the INCOM [38–42]. In particular, the reversely scored Item 11 proved demonstrated factorial instability in previous studies, loading the opinion-related factor in some [13] and the ability-related factor in others [13,37,39], whereas Item 7 showed relatively low factor loadings compared to other positively scored items [38,40,41]. These issues resulted in the removal of Item 11 from the Portuguese [41], Brazilian [42], version of the INCOM, and both Items 11 and 7 from the German [38,39] and the Spanish [40] versions of the tool. After thorough examination of the items, we concluded that the wording of these items may underlie these factorial discrepancies as they do not directly refer to the comparative process. Item 11 (*I never consider my situation in life relative to that of other people*) does not refer directly to either ability or opinion comparison but rather describes one’s experiences as not dependent on other people’s circumstances. As such it may not be the best representation of the measured construct. Similarly, Item 7 (*I often like to talk with others about mutual opinions and experiences*) refers to sharing one’s experience and views with others and thus may reflect rather conversational circumstances than comparative ones.

We first removed Item 11, which improved the model fit (∆χ2 = 57.32; ∆*df* = 9; *p* < .001). However, Item 7 continued to show high residual errors with Item 8 (MI = 49.42), Item 9 (MI = 12.32), and a cross-loading with ability comparison factor (MI = 11.33). Subsequently, we removed Item 7, which further improved the model fit (∆χ2 = 49.02; ∆*df* = 8; *p* < .001). Therefore, based on the combination of these statistical, linguistic, and theoretical premises, we decided to remove both items from further analyses.

We again tested the nine-item version of the INCOM with two factors. The goodness of fit indicated a good fit: χ^2^ = 39.36, *df* = 26, *p* = .045, χ^2^/*df* = 1.51, CFI = .99, TLI = .99, RMSEA = .033, RMSEA CI 90% [.008;.052], SRMR = .028. We then tested a one-factor model, which showed a poor fit: χ^2^ = 258.4, *df* = 27, *p* < .001, χ^2^/*df* = 9.57, CFI = .84, TLI = .79, RMSEA = .14, RMSEA CI 90% [.12;.15], SRMR = .103. The model comparison of the two-factor model to a one-factor model further supported the two-factor structure of the INCOM-PL (∆χ^2^ = 217.2; ∆*df* = 1; *p* < .001).

We then tested the multi-group CFA between men (*n* = 100) and women (*n* = 355). Due to an insufficient number of non-binary participants (*n* = 8) and participants identifying with “other” gender (*n* = 2), we had to exclude their responses from the measurement invariance analysis, given that the rule of thumb minimum number of respondents for each group in MGCFA is 100 [75]. The comparison of the changes in goodness of fit in the subsequent models indicated that the scalar measurement invariance between men and women was established (Table 2).

**Table 2:** Goodness of fit indices and model comparisons for measurement invariance models.

Model	χ^2^	*df*	CFI	TLI	RMSEA (90% CI)	SRMR	Model comparison	∆CFI	∆TLI	∆RMSEA	∆SRMR
Study 1 (*n* = 455)											
Men (*n* = 100)	34.87	26	.972	.961	.058 (.000;.103)	.061					
Women (*n *= 355)	36.47	26	.991	.988	.034 (.000;.057)	.028					
(A) Configural	71.35	52	.987	.982	.040 (.012;.061)	.035					
(B) Metric	76.57	59	.988	.985	.036 (.000;.057)	.043	B vs. A	.001	.003	−.004	.008
(C) Scalar	83.87	66	.988	.987	.034 (.000;.054)	.041	C vs. B	0	.002	−.002	−.002
Study 2 (*n* = 983)											
Men (*n *= 263)	48.81	26	.966	.953	.058 (.034;.081)	.048					
Women (*n* = 720)	79.33	26	.970	.958	.053 (.041;.066)	.040					
(A) Configural	129.12	52	.968	.956	.055 (.044;.066)	.042					
(B) Metric	149.05	59	.963	.955	.056 (.046;.066)	.053	B vs. A	−.005	−.001	.001	.011
(C) Scalar	166.79	66	.959	.955	.056 (.046;.065)	.049	C vs. B	−.004	0	0	−.004

*Note. df* = degrees of freedom, CFI = Comparative Fit Index, TLI = Tucker-Lewis Index, RMSEA = Root Mean Square Error of Approximation, SRMR = Standardized Root Mean Square Residual, ∆ change between a less restricted and a more restricted model.

### The INCOM-PL’s validity and reliability

We tested the INCOM-PL’s convergent and discriminant validity by performing a correlation analysis (Table 3). There was a strong [76], positive correlation between ability comparisons and maladaptive perfectionism (*r* = .63; *p* < .001). Ability comparisons also correlated strongly and negatively with self-compassion (*r* = −.55; *p* < .001). Opinion comparisons correlated weakly and positively with maladaptive perfectionism (*r* = .18; *p* < .001) and negatively with self-compassion (*r* = −.11; *p* = .022). However, the correlation with self-compassion was weak [76], and the correlations of both self-compassion (*z* = −7.72; *p* < .001) and maladaptive perfectionism (*z* = 8.50; *p* < .001) were stronger with ability comparisons than form opinion comparisons.

**Table 3:** Descriptive statistics and correlations in Study 1.

Variable	*M* (*SD*)	1.	2.	3.	4.	5.	6.	7.	8.	9.	10.	11.	12.
1. Ability comparisons	22.23 (5.76)	−											
2. Opinion comparisons	11.22 (2.51)	.36***	−										
3. Self-compassion (SC)	65.47 (21.33)	−.55***	−.11*	−									
4. Self-kindness (SC subscale)	12.88 (5.34)	−.38***	.02	.86***	−								
5. Self-judgement (SC subscale)	17.73 (4.88)	.51***	.18***	−.84***	−.68***	−							
6. Common humanity (SC subscale)	10.83 (4.08)	−.30***	.01	.76***	.64***	−.50***	−						
7. Isolation (SC subscale)	14.93 (3.98)	.59***	.17***	−.80***	−.54***	.68***	−.47***	−					
8. Mindfulness (SC subscale)	11.31 (3.82)	−.39***	−.02	.82***	.73***	−.51***	.65***	−.54***	−				
9. Over-identification (SC subscale)	14.89 (3.88)	.56***	.20***	−.83***	−.56***	.68***	−.51***	.74***	−.65***	−			
10. Maladaptive perfectionism	99.61 (29.16)	.63***	.18***	−.82***	−.66***	.78***	−.52***	.76***	−.56***	.72***	−		
11. Adaptive perfectionism	63.72 (13.25)	.02	.03	.05	.08	.09*	.02	−.06	.16***	−.02	.09	−	
12. Age	29.33 (9.21)	−.34***	−.13**	.26***	.19***	−.24***	.19***	−.25***	.18***	−.21***	−.33***	−.02	−

*Note.* Correlations were measured using Pearson’s **r. SD* *= standard deviation, **M* *= mean, SC = self-compassion. **N* *= 465

*p < .05, **p < .01, ***p < .001

Ability comparisons correlated positively and strongly [76] with the following self-compassion subscales: self-judgement (*r* = .51; *p* < .001), isolation (*r* = .59; *p* < .001), and over-identification (*r* = .56; *p* < .001), and negatively and moderately [76] with self-kindness (*r* = −.38; *p* < .001), common humanity (*r* = −.30; *p* < .001), and mindfulness (*r* = −.39; *p* < .001). Opinion comparisons correlated positively and weakly [76] with self-judgement (*r* = .18; *p* < .001), isolation (*r* = .17; *p* < .001), and over-identification (*r* = .20; *p* < .001), but did not correlate with the remaining three subscales. Both ability and opinion comparisons correlated negatively with age, however, the correlation was stronger (*z* = −3.40; *p* < .001) with ability comparisons (*r* = −.34; *p* < .001) than with opinion comparisons (*r* = −.13; *p* = .005). Neither opinion nor ability comparisons correlated with adaptive perfectionism.

Lastly, we calculated internal consistency coefficients for ability comparisons (α = .89, ω = .89) and opinion comparisons (α = .75, ω = .75.). Both subscales of the INCOM-PL demonstrated good internal consistency.

## Study 2

### Participants

The sample consisted of 1,001 adult participants (720 female, 263 male, 17 non-binary persons, and one person identifying as “other” gender) aged 18–86 years (*M* = 28.15; *SD* = 9.38). The majority of the participants had higher education (56.4%), followed by high-school degrees (44.1%), 1.4% had middle-school education, 0.6% had primary education, and 0.5% had vocational education. The participants declared their occupation as follows: 40.7% of the participants were working professionals, 33.7% were university students, 3.5% were school students, 18% were working and studying simultaneously 18%, 2.9% were unemployed, 0.8% were retired, and 0.4% were receiving a pension. One person did not disclose their occupational status (0.1%).

### Measures

We used the Polish nine-item version (INCOM-PL) of the Iowa Netherlands Comparison Orientation Measure (INCOM) [13] to assess social comparisons. Like previously, the scores were summed for indices of two subscales: ability comparisons and opinion comparisons. The internal consistency coefficients of ability comparisons in this study were α = .86, ω = .87, and for opinion comparisons: α = .69, ω = .70.

To measure the IP, we used the Polish version of the Brief Impostor Phenomenon Scale [77], which is based on the Perceived Fraudulence Scale [32]. The scale consists of 12 items and the scores can be summed to a general IP score and three subscales: self-deprecation, inauthenticity, and external ability attribution. The participant rate how much they agree with such statements as “I often feel I receive praise or grades that I don’t deserve” or “In general, significant people in my life tend to believe that I am more academically or professionally competent than I really am” using a scale from 1 = *strongly disagree* to 7 = *strongly agree*. The reliability of the general score in the current study was α = .88, ω = .89 and for the subscales as follows: self-deprecation (α = .80, ω = .81), inauthenticity (α = .68, ω = .70), external ability attribution (α = .81, ω = .82).

To assess the Big Five personality dimensions, we used the Polish version [78] of the 20-item Mini-IPIP scale [79]. The scores were summed for indices of five scales and their reliability in the current study was as follows: extraversion (α = .87, ω = .87), agreeableness (α = .71, ω = .73), conscientiousness (α = .80, ω = .80), emotional stability (α = .80, ω = .82), and intellect (α = .72, ω = .73). The participants respond by rating how well each of the statements such as “[I] get upset easily” or “[I] like order” described them (1 = *very inaccurate*; 5 = *very accurate*).

To measure self-esteem, we used the Polish version [80] of the Self-Liking/Self-Competence Scale-Revised (SLCS-R) [81]. The Polish version consists of 16 items, which can be summed to a total score of self-esteem (α = .91, ω = .91) and two subscales: self-liking (α = .91, ω = .91) and self-competence (α = .79, ω = .80). The internal consistency coefficients in the current study were as follows: general score (α = .91, ω = .91), self-liking (α = .91, ω = .91) self-competence (α = .79, ω = .80). Participants rate how much they agreed (from 1 = *strongly disagree* to 5 = *strongly agree*) with such statements as “I feel great about who I am” and “I am almost always able to accomplish what I try for”.

### Results

### CFA and MGCFA

We examined the factor structure of the nine-item Polish INCOM once again by testing the two-factor structure model. The goodness of fit indices indicated a good fit: χ^2^ = 108.9, *df* = 26, *p* < .001, χ^2^/*df* = 4.19, CFI = .97, TLI = .95, RMSEA = .056, (90% CI [.047;.067]), and SRMR = .039. We then conducted a multi-group CFA to examine the replicability of the INCOM-PL’s gender measurement invariance. Again, due to an insufficient number of non-binary participants (*n* = 17) and participants identifying with “other” gender (*n* = 1), we had to exclude their responses from the measurement invariance analysis. The results of the MGCFA presented in Table 2 support the establishment of scalar measurement invariance between men and women.

### Correlations

We then conducted a correlation analysis (Table 4). We found that both opinion and ability comparisons correlated positively with IP and its subscales, however, the IP correlation with ability comparisons was strong (*r* = .52; *p* < .001) [76], while with opinion comparisons was weak (*r* = .19; *p* < .001) [76]. A similar pattern occurred with the negative correlations of emotional stability with ability comparisons (*r* = −.49; *p* < .001), and with opinion comparisons (*r* = −.24; *p* < .001) and between self-esteem and ability (*r* = −.47; *p* < .001), and opinion comparisons. (*r* = −.14; *p* < .001). Like in Study 1, both ability (*r* = −.32; *p* < .001) and opinion comparisons (*r* = −.09; *p* = .005) correlated negatively with age, though the correlation with ability comparisons was stronger (*z* = −5.39; *p* < .001).

**Table 4:** Descriptive statistics and correlations in Study 2.

Variable	*M* (*SD*)	1	2.	3.	4.	5.	6.	7.	8.	9.	10.	11.	12.	13.
1. Ability comparisons	22.39 (5.29)	−												
2. Opinion comparisons	11.44 (2.29)	.34***	−											
3. Impostor phenomenon (BIPS)	53.25 (13.87)	.52***	.19***	−										
4. External ability attribution (BIPS subscale)	16.37 (5.85)	.40***	.16***	.89***	−									
5. Self-deprecation (BIPS subscale)	20.23 (5.38)	.51***	.19***	.86***	.65***	−								
6. Inauthenticity (BIPS subscale)	16.65 (4.90)	.44***	.13***	.83***	.61***	.56***	−							
7. Extraversion	10.93 (4.25)	−.15***	.12***	−.34***	−.31***	−.33***	−.22***	−						
8. Conscientiousness	11.75 (4.08)	−.11***	−.07*	−.21***	−.18***	−.14***	−.23***	.05	−					
9. Agreeableness	15.24 (3.01)	.08*	.27***	.00	.01	.04	−.06*	.29***	.04	−				
10. Emotional stability	8.95 (3.56)	−.49***	−.24***	−.56***	−.47***	−.59***	−.39***	.22***	.20***	−.05	−			
11. Intellect	15.14 (3.25)	−.19***	−.01	−.30***	−.33***	−.29***	−.14***	.27***	.00	.10**	.18***	−		
12. Self-esteem	41.60 (12.46)	−.47***	−.14***	−.73***	−.66***	−.69***	−.52***	.36***	.26***	.00	.65***	.36***	−	
13. Age	28.15 (9.78)	−.32***	−.09**	−.24***	−.18***	−.28***	−.14***	.12***	.08**	−.02	.23***	.16***	.22***	−

*Note.* Correlations were measured using Pearson’s **r. SD* *= standard deviation, **M* *= mean, BIPS = Brief Impostor Phenomenon Scale.

**N* *= 1,001

*p < .05, **p < .01, ***p < .001

### Indirect effect model

**Fig 1:**
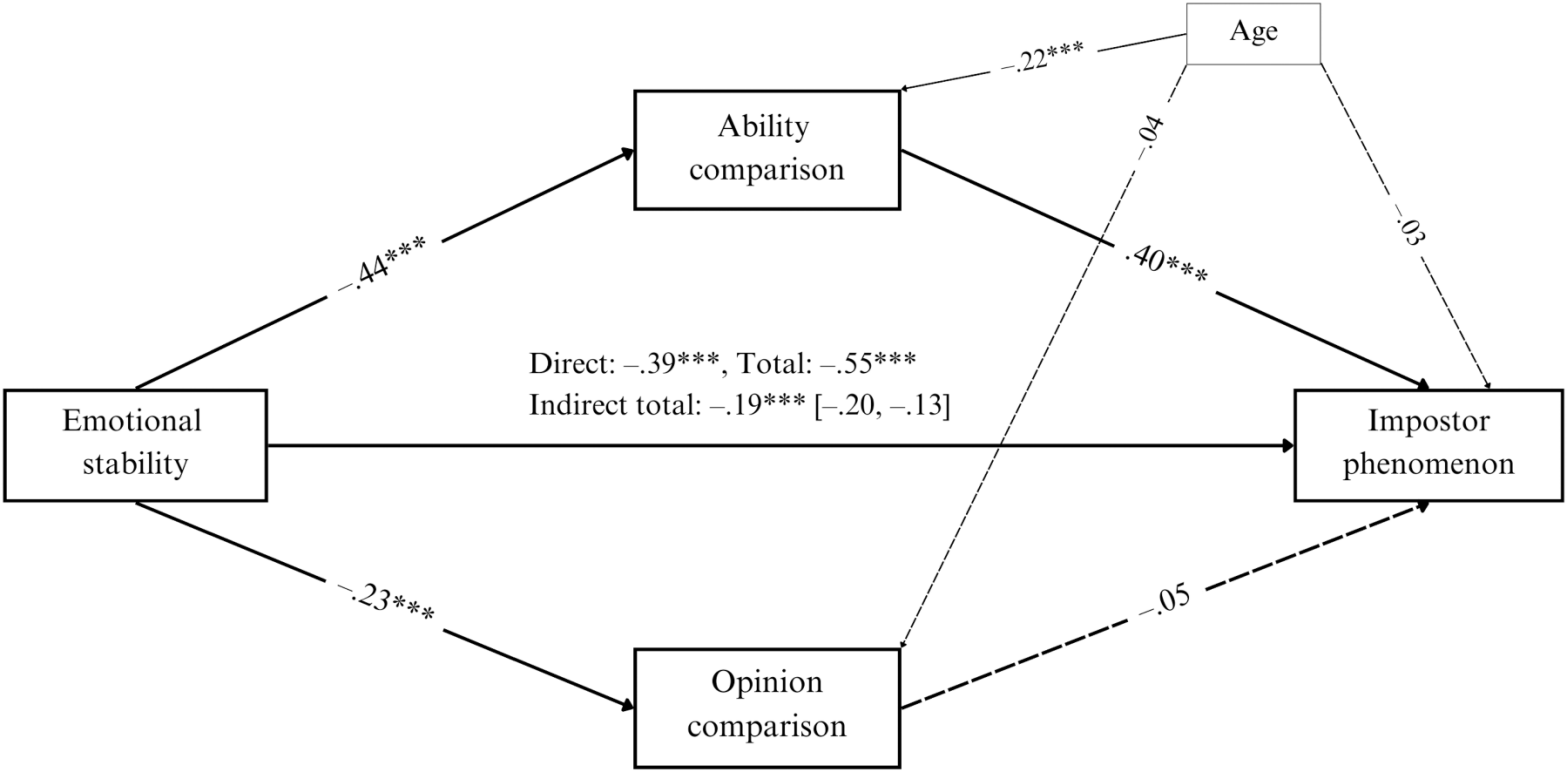
Indirect effect model with ability and opinion comparisons as intervening variables in the relationship between emotional stability and the impostor phenomenon. The model presents standardized coefficients. Continuous lines represent significant relationships and dotted lines – non-significant. The model was adjusted for age. *** *p* < .001; *N* = 1,001.

Lastly, we tested the indirect effect of ability comparisons and opinion comparisons (Fig 1) in the relationship between emotional stability and the IP. The overall model (*F*(4; 996) = 209.77, *p* < .001) explained 46% of IP’s variance. The negative direct effect of emotional stability on the IP was significant (β = −.39, *SE* = .03, *p* < .001, 95% CI [−.44, − .34]). The reduction in the standardized beta coefficient (c = −.58; c’ = −.39) and the significant completely standardized indirect effect (*ab*_*cs*_ = −.18, *SE* = 0.02, 95% CI [−.21, − .14]) suggest a partial indirect effect of ability comparisons. The indirect effect size was medium [70]. There was no indirect effect of opinion comparisons in the relationship between emotional stability and the IP (*ab*_*cs* _= −.01, *SE* = .01, 95% CI [−.00,.02]). The partial indirect effect of ability comparisons in the relationship between emotional stability and the IP suggests that people with low emotional stability tend to frequently compare their abilities with others, which may, in turn, be related with higher levels of the IP.

### Conditional process analysis

Following the indirect effect model, we then tested whether the indirect effects were conditional on gender (women were coded as 1 and men as 0) using Model 59 in the PROCESS plug-in [67]. Gender as a moderator was tested on all paths: a and b (indirect effect), and c (direct effect). We found no conditional indirect effect neither for ability comparisons (*ab*_*cs*_ = −.59; *SE* = .04, 95% CI [−.13;.01]) nor opinion comparisons (*ab*_*cs* _= .00; *SE *= .02, 95% CI [−.04;.03]) on either one of these paths. The indirect and the direct effects for each gender are presented in S2 Table. This suggests that the indirect effect of ability comparisons occurred regardless of the participants’ gender.

## Discussion

The first purpose of the current study was to introduce a Polish version of the INCOM for measuring social comparisons and test its factorial, convergent, and discriminant validity. The confirmatory factor analysis (CFA) supported the originally proposed [13] two-factor structure of the INCOM-PL, distinguishing between ability and opinion comparisons. However, some items had very weak loadings (in particular, Item 11), and high modification indices (Item 7) due to potential issues with their wording, therefore supporting their removal. These items also proved problematic in other language versions of the INCOM [38–42]. This refinement resulted in a robust nine-item version that had good internal consistency and validity in two independent samples. The MGCFA between genders also indicated that scalar measurement invariance was established between men and women, suggesting that the INCOM measures psychometrically equivalent construct in these groups, and therefore enables making meaningful comparisons between means.

We validated the INCOM-PL by replicating the previously established correlations of social comparisons. In line with previous findings [16,26–28], both ability and opinion comparisons positively correlated with maladaptive perfectionism and negatively with self-compassion, though opinion comparisons showed weaker associations with these constructs. These findings suggest that people high in perfectionism, who set unrealistically high expectations for themselves, may be especially self-critical about their self-perceived shortcomings when comparing their abilities with the achievements of others. Similarly, highly self-compassionate people, with a kind and understanding approach to their flaws, do not seem to need to compare their abilities with others to feel secure and satisfied with themselves. Furthermore, we found that ability comparisons demonstrated strong, positive correlations with self-judgement, isolation, and overidentification — considered as negative facets of self-compassion related to self-coldness [73] — and moderate, negative ones with the positive ones: self-kindness, common humanity, and over-identification. Contrastingly, opinion comparisons correlated weakly only with the three subscales representing self-coldness, but not with the positive ones. This finding further supports the notion that the adverse mental health outcomes related to frequent social comparisons may be driven solely by the competition-based ability comparisons [44].

Additionally, in Study 2, we replicated the previously found [13] negative correlations between social comparisons and self-esteem and emotional stability (i.e., low neuroticism). Finally, in both of our studies, in line with the findings of Buunk et al. [46] and Callan et al. [47], the older people were, the less they compared their abilities and opinions with others, though this negative correlation was stronger for ability comparisons. These findings suggest that young adults, with newly gained independence and still in the process of forming their identity, may be more eager to seek information about how their peers are doing, while older individuals may self-assess based on their own past self rather than other their age [47]. The weaker association of age with opinion-related comparisons may be explained by opinion comparisons’ links with agreeableness — which raises with age [82] — suggesting that more mature people are also more inclined to evaluate their beliefs and opinions in relation to those of other peoples’.

Our findings shed new light on the well-established links between neuroticism (i.e., low emotional stability) and the IP [49,50] by highlighting the role of social comparisons in this relationship, particularly those that are performance-related. The results of our research suggest a potential mechanism, which may underlie the development of self-perceived intellectual fraudulence in people high in neuroticism through frequent comparisons of their abilities. These effects were independent of participants’ gender. The lack of indirect effect of opinion comparisons suggests that the IP is driven by performance-related comparisons and one’s competence insecurities rather than discrepancies in personal beliefs or values. Along with the previously described factor analysis, these findings also suggest that ability and opinion comparisons are related, yet distinct constructs [13,37,39] rather than a unidimensional one [40,41]. Our studies highlight the potential advantage of distinguishing between these two facets instead of relying solely on the total score of social comparison orientation, considering that this distinction may offer deeper insight into various complex psychological mechanisms, that would otherwise be overlooked.

Furthermore, revealing this indirect effect of ability comparisons in the relationship between emotional stability and the IP may have some practical implications. Brief online self-compassion-based intervention showed promise in reducing the IP [83], though still, to date, therapeutic interventions aimed at minimizing the IP are limited and focus rather on IP’s negative psychological outcomes or comorbidities [84]. Social comparisons are a process that could be suspectable to some forms of modification [85], for example, through various cognitive-behavioral therapy-based practices such as cognitive restructuring [86]. Group interventions have already proven successful in minimizing social comparison concern, that is concern over one’s performance relative to others [87]. Thus, given the strong, negative correlations between self-compassion and social comparisons, reducing the urge to compare one’s abilities with others by fostering a kind and non-judgmental attitude towards self through self-compassion-based interventions may also prove useful in preventing the development of the IP. Further research in this area is warranted.

## Limitations and conclusions

Several limitations should be acknowledged when interpreting the results of our research. First, it is crucial to note that both of our studies were cross-sectional, therefore making it impossible to draw definitive causal inferences or temporal conclusions [88] about the directions of the relationships between emotional stability, social comparisons, and the IP in our indirect effect and conditional indirect effect models. While our models are supported by previous research [31,53], longitudinal studies are needed to further test whether social comparison tendencies develop in response to low emotional stability or actively contribute to the IP over time. Additionally, even though the Mini-IPIP is a reliable and widely used measure [89,90], it is worth noting that it uses only four items for assessing each of personality traits, including emotional stability. Therefore, it is advised that future studies examine our model in a longitudinal manner, preferably using a more refined measure designed specifically for assessing emotional stability.

Second, our studies relied solely on Polish samples recruited through online methods, which may limit the generalizability to other cultural contexts, especially considering that social comparison processes may differ across cultures [91]. Lastly, our participants’ gender in both samples was not equally distributed, with the majority of the participants being female (76% in Study 1 and 72% in Study 2) and having higher education. Even though overrepresentation of female participants and those with higher education is common in voluntary samples [92,93]. This limitation is important to note when interpreting the lack of moderating effect of gender as well as gender invariance findings. While in Study 2 both group sizes were optimal, Study 1 included 100 male respondents. Even though the rule of thumb sample size per group in MGCFA is 100 [75], and studies often include groups of 100 or fewer participants [94,95], a larger group size provides more precise estimates and more reliable invariance tests. Future studies should aim to replicate our findings in a more gender-representative samples.

Despite these limitations, our research contributes to the current literature by presenting a Polish version of the Iowa-Netherlands Comparison Orientation Measure (INCOM-PL) with robust psychometric properties and by offering insight into correlates and mechanisms related to social comparisons and the IP. Our findings highlight the indirect role of ability comparisons in the relationship between emotional stability and the IP, emphasizing the importance of self-evaluation processes, particularly performance-related, in shaping self-doubt and self-perceived intellectual fraudulence. Future studies, ideally with a longitudinal design, should focus on testing the applicability of our findings by examining the potentially beneficial effects of targeting maladaptive patterns of social comparison in interventions aimed at reducing IP.

## Supporting information

S1 Table Descriptive statistics of the full Polish version of the INCOM in Study 1. *Note.* *items were not included in the INCOM-PL. *M* = mean; *SD* = standard deviation. The full version of the INCOM-PL can be found can be found in the Supporting information S3 File. *N* = 465.
(DOCX)

S2 Table Indirect effects for each gender in the conditional indirect effect model. *Note*. *SE* = standard error, LLCI = lower-level confidence interval, ULCI = upper-level confidence interval. The coefficients are standardized. Age was controlled for in the conditional indirect effect model. Women: *n* = 720; men: *n* = 263.
(DOCX)

S3 File The full Polish version of the INCOM. (DOCX)

S4 File The original English version of the INCOM proposed by Gibbons and Buunk (1999). (DOCX)

S5 File Database from Study 1. (XLSX)

S6 File Database from Study 2. (XLSX)
