# Supplementary material for: Is every comparison a thief of joy? Polish validation of the Iowa-Netherlands Comparison Orientation Measure and the indirect role of social comparisons in the relationship between emotional stability and the impostor phenomenon
Source: PLoS One. 2025 Sep 25;20(9):e0333095. doi: 10.1371/journal.pone.0333095 (PMC12463242; doi:10.1371/journal.pone.0333095)
Supplement: S1 Table — Note. *items were not included in the INCOM-PL. M = mean; SD = standard deviation. The full version of the INCOM-PL can be found can be found in the Supporting information S3 File. N = 465. (DOCX) [file pone.0333095.s001.docx]

**S1 Table**. **Descriptive statistics of the full Polish version of the INCOM in Study 1.**

| Item number | Item | *M* | *SD* | Skewness | Kurtosis |
| --- | --- | --- | --- | --- | --- |
| 1 | I often compare how my loved ones (boy or girlfriend, family members, etc.) are doing with how others are doing./*Często porównuję, jak radzą sobie moi bliscy (partner lub partnerka, członkowie rodziny itp.) w porównaniu z innymi.* | 3.30 | 1.26 | −0.32 | −1.04 |
| 2 | I always pay a lot of attention to how I do things compared with how others do things./*Zawsze zwracam szczególną uwagę na to, jak ja robię pewne rzeczy w porównaniu z tym, jak robią to inni.* | 3.75 | 1.16 | −0.81 | −0.33 |
| 3 | If I want to find out how well I have done something, I compare what I have done with how others have done./*Jeśli chcę się dowiedzieć, jak dobrze coś zrobiłem(-am), porównuję swoje wykonanie z tym, jak zrobili to inni.* | 3.82 | 1.01 | −0.84 | -0.04 |
| 4 | I often compare how I am doing socially (e.g., social skills, popularity) with other people./*Często porównuję to, jak radzę sobie w sytuacjach społecznych (np. umiejętności społeczne, popularność) z tym, jak radzą sobie inni ludzie.* | 3.85 | 1.19 | −0.93 | −0.07 |
| 5 | I am not the type of person who compares often with others. (reversed item)/*Nie jestem typem osoby, która porównuje się z innymi. (item odwrócony)* | 3.85 | 1.21 | −0.87 | −0.22 |
| 6 | I often compare myself with others with respect to what I have accomplished in life./*Często porównuję się z innymi pod względem tego, co osiągnąłem(-am) w życiu.* | 3.67 | 1.26 | −0.70 | −0.60 |
| 7 | I often like to talk with others about mutual opinions and experiences.*/*Często lubię rozmawiać z innymi na temat wspólnych poglądów i doświadczeń.** | 4.00 | 0.98 | −1.01 | 0.73 |
| 8 | I often try to find out what others think who face similar problems as I face./*Często staram się dowiedzieć, co myślą osoby mierzące się z podobnymi problemami, co ja.* | 3.91 | 0.98 | −0.95 | 0.58 |
| 9 | I always like to know what others in a similar situation would do./*Zawsze lubię wiedzieć, jak na moim miejscu postąpiliby inni.* | 3.55 | 1.10 | −0.52 | −0.54 |
| 10 | If I want to learn more about something, I try to find out what others think about it./*Kiedy chcę dowiedzieć się czegoś więcej na jakiś temat, próbuję się dopytać, jakie jest na ten temat zdanie innych.* | 3.76 | 0.99 | −0.79 | 0.22 |
| 11* | I never consider my situation in life relative to that of other people. (reversed item)*/*Nigdy nie uważam mojej sytuacji życiowej za zależną od innych ludzi. (item odwrócony)** | 3.15 | 1.12 | −0.14 | −0.76 |

Note. *items were not included in the INCOM-PL. M = mean; SD = standard deviation. The full version of the INCOM-PL can be found in the Supporting information S3 File. N = 465
