# Supplementary material for: Is every comparison a thief of joy? Polish validation of the Iowa-Netherlands Comparison Orientation Measure and the indirect role of social comparisons in the relationship between emotional stability and the impostor phenomenon
Source: PLoS One. 2025 Sep 25;20(9):e0333095. doi: 10.1371/journal.pone.0333095 (PMC12463242; doi:10.1371/journal.pone.0333095)
Supplement: S2 Table — Note. SE = standard error, LLCI = lower-level confidence interval, ULCI = upper-level confidence interval. The coefficients are standardized. Age was controlled for in the conditional indirect effect model. Women: n = 720; men: n = 263. (DOCX) [file pone.0333095.s002.docx]

**S2 Table**. **Indirect effects for each gender in the conditional indirect effect model.**

| Path | | Effect | *SE* | 95% LLCI | 95% ULCI |
| --- | --- | --- | --- | --- | --- |
| *Emotional stability → Ability comparison → Impostor phenomenon* | | | | | |
|  | Men | –.14 | .03 | –.19 | –.09 |
|  | Women | –.19 | .02 | –.24 | –.15 |
| *Emotional stability → Opinion comparison → Impostor phenomenon* | | | | | |
|  | Men | .01 | .02 | –.02 | .04 |
|  | Women | .01 | .01 | .00 | .02 |
| *Direct effect of Emotional stability on Impostor phenomenon* | | | | | |
|  | Men | –.39 | .05 | –.49 | –.30 |
|  | Women | –.39 | .03 | –.46 | –.33 |

*Note*. *SE* = standard error, LLCI = lower-level confidence interval, ULCI = upper-level confidence interval. The coefficients are standardized. Age was controlled for in the conditional indirect effect model. Women: *n* = 720; men: *n* = 263
