# Supplementary material for: Is every comparison a thief of joy? Polish validation of the Iowa-Netherlands Comparison Orientation Measure and the indirect role of social comparisons in the relationship between emotional stability and the impostor phenomenon
Source: PLoS One. 2025 Sep 25;20(9):e0333095. doi: 10.1371/journal.pone.0333095 (PMC12463242; doi:10.1371/journal.pone.0333095)
Supplement: S3 File — (DOCX) [file pone.0333095.s003.docx]

**S3 File. The full Polish version of the INCOM.**

**Skala Porównań Społecznych INCOM-PL**

| Większość ludzi porównuje się z innymi od czasu do czasu. Przykładowo, mogą porównywać to, jak się czują, jakie mają opinie, zdolności i/lub sytuacje życiowe z innymi ludźmi. Nie ma nic „złego” lub „dobrego” w takich porównaniach, a niektórzy angażują się w nie częściej niż inni.  Chcielibyśmy dowiedzieć się, jak często Ty porównujesz się z innymi. Aby to sprawdzić, oceń, proszę, na ile zgadzasz się z każdym z poniższych stwierdzeń, posługując się skalą od 1 (zdecydowanie się nie zgadzam) do 5 (zdecydowanie się zgadzam). | | zdecydowanie się nie zgadzam | nie zgadzam się | ani się nie zgadzam, ani zgadzam | zgadzam się | zdecydowanie się zgadzam |
| --- | --- | --- | --- | --- | --- | --- |
| 1 | Często porównuję, jak radzą sobie moi bliscy (partner lub partnerka, członkowie rodziny itp.) w porównaniu z innymi. | 1 | 2 | 3 | 4 | 5 |
| 2 | Zawsze zwracam szczególną uwagę na to, jak ja robię pewne rzeczy w porównaniu z tym, jak robią to inni. | 1 | 2 | 3 | 4 | 5 |
| 3 | Jeśli chcę się dowiedzieć, jak dobrze coś zrobiłem(-am), porównuję swoje wykonanie z tym, jak zrobili to inni. | 1 | 2 | 3 | 4 | 5 |
| 4 | Często porównuję to, jak radzę sobie w sytuacjach społecznych (np. umiejętności społeczne, popularność) z tym, jak radzą sobie inni ludzie. | 1 | 2 | 3 | 4 | 5 |
| 5 | Nie jestem typem osoby, która porównuje się z innymi. (item odwrócony) | 1 | 2 | 3 | 4 | 5 |
| 6 | Często porównuję się z innymi pod względem tego, co osiągnąłem(-am) w życiu. | 1 | 2 | 3 | 4 | 5 |
| 8 | Często staram się dowiedzieć, co myślą osoby mierzące się z podobnymi problemami, co ja. | 1 | 2 | 3 | 4 | 5 |
| 9 | Zawsze lubię wiedzieć, jak na moim miejscu postąpiliby inni. | 1 | 2 | 3 | 4 | 5 |
| 10 | Kiedy chcę dowiedzieć się czegoś więcej na jakiś temat, próbuję się dopytać, jakie jest na ten temat zdanie innych. | 1 | 2 | 3 | 4 | 5 |

Porównania umiejętności: 1, 2, 3, 4, 5, 6

Porównania opinii: 8, 9, 10

Item 7 wykluczony z INCOM-PL: Często lubię rozmawiać z innymi na temat wspólnych poglądów i doświadczeń.

Item 11 wykluczony z INCOM-PL: Nigdy nie uważam mojej sytuacji życiowej za zależną od innych ludzi. (item odwrócony)
