# Supplementary material for: Is every comparison a thief of joy? Polish validation of the Iowa-Netherlands Comparison Orientation Measure and the indirect role of social comparisons in the relationship between emotional stability and the impostor phenomenon
Source: PLoS One. 2025 Sep 25;20(9):e0333095. doi: 10.1371/journal.pone.0333095 (PMC12463242; doi:10.1371/journal.pone.0333095)
Supplement: S4 File — (DOCX) [file pone.0333095.s004.docx]

**S4 File. The original English version of the INCOM proposed by Gibbons and Buunk (1999).**

**Iowa-Netherlands Comparison Orientation Measure**

| Most people compare themselves from time to time with others. For example, they may compare the way they feel, their opinions, their abilities, and/or their situation with those of other people. There is nothing particularly ‘good’ or ‘bad’ about this type of comparison, and some people do it more than others.  We would like to find out how often you compare yourself with other people. To do that we would like to ask you to indicate how much you agree with each statement below, by using the following scale:  1 = I disagree strongly to 5 = I agree strongly | | 1 = I disagree strongly | 2 | 3 | 4 | 5 = I agree strongly |
| --- | --- | --- | --- | --- | --- | --- |
| 1 | I often compare how my loved ones (boy or girlfriend, family members, etc.) are doing with how others are doing. | 1 | 2 | 3 | 4 | 5 |
| 2 | I always pay a lot of attention to how I do things compared with how others do things. | 1 | 2 | 3 | 4 | 5 |
| 3 | If I want to find out how well I have done something, I compare what I have done with how others have done. | 1 | 2 | 3 | 4 | 5 |
| 4 | I often compare how I am doing socially (e.g., social skills, popularity) with other people. | 1 | 2 | 3 | 4 | 5 |
| 5 | I am not the type of person who compares often with others. (*reversed item*) | 1 | 2 | 3 | 4 | 5 |
| 6 | I often compare myself with others with respect to what I have accomplished in life. | 1 | 2 | 3 | 4 | 5 |
| 7 | I often like to talk with others about mutual opinions and experiences. | 1 | 2 | 3 | 4 | 5 |
| 8 | I often try to find out what others think who face similar problems as I face. | 1 | 2 | 3 | 4 | 5 |
| 9 | I always like to know what others in a similar situation would do. | 1 | 2 | 3 | 4 | 5 |
| 10 | If I want to learn more about something, I try to find out what others think about it. | 1 | 2 | 3 | 4 | 5 |
| 11 | I never consider my situation in life relative to that of other people. (*reversed item*) | 1 | 2 | 3 | 4 | 5 |

*Note.* Items 7 and 11 were not included in the Polish version of the INCOM (INCOM-PL). The INCOM-PL can be found in the Supporting information S3 File.

Ability comparisons: items 1- 6; Opinion comparisons: items 7-11
